# High-throughput circular RNA sequencing reveals the profiles of circular RNA in non-cirrhotic hepatocellular carcinoma

**DOI:** 10.1186/s12885-022-09909-2

**Published:** 2022-08-05

**Authors:** Hongyu Li, Liangliang Xu, Pengsheng Yi, Lian Li, Tao Yan, Liang Xie, Zhijun Zhu

**Affiliations:** 1 Department of Liver Transplantation, Beijing Friendship Hospital, Capital Medical University, Beijing, 100050 China; 2 Department of Liver Surgery and Liver Transplantation Center, West China Hospital, Sichuan University, Chengdu, 610041 China; 3 Department of Hepato-Biliary-Pancreas, Affiliated Hospital of North Sichuan Medical College, Nanchong, 637000 Sichuan Province China

**Keywords:** Hepatocellular carcinoma, Non-cirrhosis, Circular RNA, Competing endogenous RNA, Coding peptides

## Abstract

**Background:**

Liver cirrhosis is a well-known risk factor for hepatocellular carcinoma (HCC). However, some HCC cases can also originate from non-cirrhotic livers. The aim of this study was to identify key circular RNAs (circRNAs) associated with the tumorigenesis of non-cirrhotic liver disease.

**Methods:**

The differently expressed circRNAs between non-cirrhotic and cirrhotic HCCs were assessed with use of high-throughput circRNAs sequencing and validated with quantitative reverse transcription polymerase chain reaction (qRT-PCR). Potential biological functions of these dysregulated circRNAs were predicted with use of Gene Ontology (GO) and Kyoto Encyclopedia of Genes and Genomes (KEGG) analyses. A circRNA-miRNA-mRNA regulation network was constructed as achieved with use of miRanda software and visualized using Cytoscape software. Biological functions of the four most prominent dysregulated circRNAs identified were confirmed by in vitro experiments. Moreover, possible translations of these dysregulated circRNAs were also predicted.

**Results:**

A total of 393 dysregulated circRNAs were identified between non-cirrhotic and cirrhotic HCC, including 213 that were significantly up-regulated and 180 significantly down-regulated circRNAs. Expression levels of the six most prominent dysregulated circRNAs were further validated using qRT-PCR. Many tumor related miRNAs were involved in the circRNA-miRNA-mRNA networks, including miR-182-5p, miR-561-3p, miR-125a-5p, miR-145, miR-23b-3p and miR-30e-3p, and downstream mRNAs of dysregulated circRNAs were significantly related with biological processes involved in the progression of tumors, including proliferation, migration, differentiation, and focal adhesion. Results from the in vitro experiments demonstrated that the most prominent dysregulated circRNAs exerted notable effects upon the proliferation and migration of HCC cells. Finally, we also identified 19 dysregulated circRNAs having potential for the coding of functional peptides.

**Conclusion:**

The results of this present study indicate that circRNAs may play important roles in tumorigenesis of non-cirrhotic HCC. Such findings provide some novel insights and pave the way for the development of future studies directed at investigating the initiation and treatment of HCC.

**Supplementary Information:**

The online version contains supplementary material available at 10.1186/s12885-022-09909-2.

## Background

Hepatocellular carcinoma (HCC) represents the most frequent pathological type of primary liver cancer. Globally, approximately 782,000 new cases are diagnosed as HCC, with about 746,000 deaths resulting from HCC each year [1]. With its characteristics of being asymptomatic at early stages, rapid growth and a tendency for intrahepatic metastasis, most HCC cases are only diagnosed at advanced stages and thus chances for curative treatment are often squandered [2]. Unfortunately, even in HCC patients receiving curative treatments such as radiofrequency ablation (RFA), partial liver resection or liver transplantation, the 5-year cumulative risk of recurrence remains at greater than 70% [3]. Therefore, identification of molecular mechanisms involved in the tumorigenesis and progression of HCC, along with the detection of effective treatment targets are imperative to improve the survival outcomes of HCC patients.

Nearly all HCC cases result from chronic liver injury, attributable to various etiologies such as hepatitis viral infection, alcohol abuse and non-alcoholic fatty liver disease (NAFLD). Chronic liver injury destroys normal liver cells and structure of the liver [4], with the subsequent regenerative nodules and fibrosis then contributing to liver cirrhosis. Almost 80% of HCC patients have a history of cirrhosis [5, 6]. Therefore, chronic liver injury, liver cirrhosis and HCC are considered as sharing a common pathological bases for the tumorigenesis of HCC. However, approximately 10–20% of HCC cases originate from non-cirrhotic livers [7, 8]. When comparing patients with cirrhotic versus non-cirrhotic HCC, the latter are usually older, more frequently females, display lower AFP levels, show an overall better liver function, more frequently underwent anatomical resection, have a lower prevalence of type II diabetes (T2DM), but have larger tumors [8, 9]. However, the molecular mechanisms underlying the transformation from non-cirrhotic liver to HCC remain unknown.

CircRNA refers to a large class of RNAs that are produced by a non-canonical splicing event called backsplicing, a process which involves a downstream splice-donor site that is covalently linked to an upstream splice-acceptor site [10]. Although circRNAs were initially observed in pathogens over 40 years ago [11], due to the absence of 3′ polyadenylated tails, it was not possible to detect most circRNAs using classical RNA sequencing (RNA-seq) datasets. Therefore, for a long time, circRNAs were considered as the ‘junk’ generated by aberrant splicing, and the biogenesis and characteristics of circRNAs were only reported in sporadic studies [12–15]. However, with the recent development of non-polyadenylated and RNase R-treated transcriptome analyses [16, 17], an abundant number of circRNAs were found to be present in metazoans [18–21], protists [22] and plants [23]. And results from an increasing array of studies have demonstrated that some circRNAs play important roles in various diseases [10, 24]. Moreover, circRNAs have been shown to regulate gene expression by modulating transcription and splicing, titrating microRNAs (miRNAs), interacting with proteins and acting as templates for the translation of polypeptides [24].

Recently, using high throughput circRNA microarray or sequencing, a number of differently expressed circRNAs have been identified between HCC and paired non-tumorous tissues. Further validation using quantitative reverse transcription polymerase chain reaction (qRT-PCR) verified that some of these circRNAs could be used as the novel biomarkers for HCC diagnosis and prognosis prediction [25–28]. Moreover, results from in vivo and in vitro experiments verified that some circRNAs play important roles in HCC tumorigenesis and progression by regulating various biological processes, including cell proliferation, migration, invasion and metastasis, epithelial-mesenchymal transition (EMT), as well as apoptosis [29–32]. However, the expression pattern and possible functions of circRNAs in the tumorigenesis of HCC as observed in non-cirrhotic livers remain unclear. In the current study, RNase R-treated transcriptome sequencing was performed to detect the presence of differently expressed circRNAs between cirrhotic and non-cirrhotic HCCs. In addition, the possible function and underlying mechanism of key circRNAs were predicted as based on this information.

## Methods

### Patients and HCC samples

Clinical samples used in the present study were obtained from the West China Hospital, of Sichuan University and the Beijing Friendship Hospital of Capital Medical University. Use of clinical specimens was approved by the Biomedical Ethics Committee of the West China and Beijing Friendship Hospitals, and written informed consent was obtained from each patient. In total, 20 HCC and paired non-tumorous tissues were selected for analyses in the present study. Histological diagnosis of HCC was performed by two professional pathologists. Fibrosis was staged according to the Ishak fibrosis scale with values ranging from 0 to 6 [33]. Patients with Ishak scores of 6 (definite cirrhosis) were allocated into the cirrhotic group, while those with Ishak scores of ≤ 3 were assigned to the non-cirrhotic group. None of the enrolled patients had any history of prior treatments such as surgery, transcatheter arterial chemoembolization (TACE), radiotherapy or chemotherapy. After matching, as based on some critical variables including age, gender, alpha fetoprotein (AFP) loading and tumor size, six HCC cases (3 originating from non-cirrhotic and 3 from cirrhotic livers) were identified for use in RNase R-treated circRNA sequencing (circRNA-seq), and all samples were used to validate the circRNA-seq results.

### High throughput circRNA sequencing

All procedures involving circRNA-seq and raw data processing were conducted by a professional company (Novogene, Beijing, China) and are briefly described below.

### Library preparation for circRNA sequencing

Total RNA was extracted from HCC tissue samples using TRIzol reagent according to the manufacturers’ instructions. RNA degradation and contamination were monitored on 1% agarose gels, while RNA concentrations and purity were assessed using the NanoPhotometer® spectrophotometer (IMPLEN, CA, USA). A total of 5 μg high-quality RNA per sample was used as input material for RNA sample preparations. Ribosomal and linear RNAs were removed using the Epicentre RibozeroTM rRNA Removal (Epicentre, USA) and RNase R (Epicentre, USA) kits, respectively. Subsequently, circRNA was enriched using acid phenol/chloroform (pH 4.5). Sequencing libraries were prepared with use of the NEBNext® UltraTM Directional RNA Library Prep Kit for Illumina® (NEB, USA) following the manufacturers’ recommendations. Briefly, fragmentation was performed using divalent cations under elevated temperatures in NEBNext First Strand Synthesis Reaction Buffer (5X). First strand cDNA was synthesized using a random hexamer primer and M-MuLV Reverse Transcriptase (RNase H). Second strand cDNA synthesis was subsequently performed using DNA Polymerase I and RNase H. In the reaction buffer, dNTPs with dTTP were replaced by dUTP. Remaining overhangs were converted into blunt ends via exonuclease/polymerase activities. After adenylation of 3’ ends of DNA fragments, the NEBNext Adaptor with hairpin loop structures were ligated to prepare for hybridization. In order to preferentially select cDNA fragments of 250–300 bp in length, library fragments were purified with use of the AMPure XP system (Beckman Coulter, Beverly, USA). Then, 3 μl of USER Enzyme (NEB, USA) was used with a size-selected, adaptor-ligated cDNA at 37 °C for 15 min followed by 5 min at 95 °C before PCR. PCR was then performed with Phusion High-Fidelity DNA polymerase, Universal PCR primers and Index (X) Primer. The products were purified (AMPure XP system) and library quality was assessed on the Agilent Bioanalyzer 2100 system. Finally, libraries were sequenced on an Illumina platform (Illumina, San Diego, CA, USA) with a pair end of 150 bp reading length.

Raw data of the fastq format were initially processed through in-house perlscripts. In this step, clean data (clean reads) were obtained by removing reads containing adapter, reads on containing uncertain nucleotide more than 0.2%, and low-quality reads which contain low quality nucleotides more than 50% from raw data. At the same time, error rate distribution along reads and GC content of the clean data were calculated. All the downstream analyses were based on the clean data with high quality. Tophat2 software (v2.0.13) was employed to align clean reads with the human reference genome (GRCh38/hg38) [34].

Sequencing data failing to directly aligned with a reference genome were subjected to subsequent circRNA analysis by recognition of the reverse splicing event. Find_ circ [35] is a basic tool for identifying circRNAs, which extracts 20 nt anchor sequences from both ends of the reads that are not aligned to the reference sequence. Then align each pair of anchor sequences to the reference sequence again. If the 5' end of the anchor sequence is aligned to the reference sequence (the start and end sites are marked as A3 and A4 respectively), meanwhile, the 3' end of the anchor sequence is aligned upstream of this site (the start and end sites are marked as A1 and A2 respectively), and there is a splice site (GT-AG) between A2 and A3 of the reference sequence, this read is considered as a candidate circRNA. Furthermore, the candidate circRNAs were also recognized using the CIRI (circRNA identifier) [36]. In brief, paired chiastic clipping, paired-end mapping, and GT-AG splicing signals were discovered via scanning the above obtained unmapped reads. Next, the alignment files were scanned again using a dynamic programming algorithm for detecting additional junction reads and eliminating false positive circRNA candidates. The final circRNAs were obtained by retaining sequences with ≥ 2 junction reads. The DESeq2 R package (2.15.13) was employed to analyze differently expressed circRNAs between cirrhotic and non-cirrhotic HCCs [37]. The original scripts of DESeq2, pheatmap and volcano visualization were contained in Additional file 1: Supplementary materials. Functional annotation and pathway enrichment analyses for host genes of differently expressed circRNAs were performed using KOBAS 2.0 [38–41].

### Validation of candidate circRNA expression

Total RNA was isolated from samples using TRIzol reagent (Life Technologies, Carlsbad, CA) and RNA integrity was evaluated in 1% agarose gels. Concentrations and purity of RNA were measured with use of a ScanDrop Nuclear Acid Analyzer (Analytik Jena AC, Jena, Germany). Removal of contaminated genome DNA (gNDA) and reverse transcription of complement DNA (cDNA) were performed using the HiScript III RT SuperMix for PCR (Vazyme Biotech Co., Ltd, Nanjing, China) according to the manufacturers’ instructions. Genomic DNA (gDNA) was extracted from HCC tissues using a PureLinkTM Genomic DNA Mini Kit (Thermo Fisher Scientific, USA), according to the manufacturers’ protocol. All primers used in this study were designed in primer 5.0 software (Premier, Canada) and synthesized by TsingKe Biotech (Chengdu, China) (Table 1). The qRT-PCR was performed using a 2 × ChamQ Universal SYBR qPCR Master Mix (Vazyme Biotech Co., Ltd) with β-actin as the internal control. Relative expressions of candidate circRNAs were calculated using the 2^−ΔΔCt^ method.

**Table 1:** All primers used in this study

Gene	Primer	Sequence (5′–3′)
hsa_circ_0002473-divergent	Forward primer	TGGACTTCACTGCAGCAAGATT
	Reverse primer	GTGCAGCTTTTGATTTGCCC
novel_circ_0013894-divergent	Forward primer	AAATCACATCAGGCTCATCAAA
	Reverse primer	CGACCTTGGGCTCAAATACT
novel_circ_0015002-divergent	Forward primer	CTGCAGTCATGAGCCTTCCT
	Reverse primer	GGCTGTAGATCGGAGGACAC
hsa_circ_0002468-divergent	Forward primer	ACAACGAGCTAATGACTTGG
	Reverse primer	AGGTTTTAAGCCATGCATCA
hsa_circ_0004524-divergent	Forward primer	CGAGCAGCCAATCAAGAAC
	Reverse primer	GGGCTACCGGAAACATAGTT
hsa_circ_0003823-divergent	Forward primer	AGCAGAAAACTTTACAGGCA
	Reverse primer	GGGCTACCGGAAACATAGTT
hsa_circ_0002473-covergent	Forward primer	TTGCTTATTATCGGAGGGCTAC
	Reverse primer	TTGCTGCAGTGAAGTCCATC
novel_circ_0013894-covergent	Forward primer	ACAATGCCAAAGCATTCTCC
	Reverse primer	TGGATCTCATCGCAGTTTGA
novel_circ_0015002-covergent	Forward primer	ATTGACAGTTTCGCCGACAT
	Reverse primer	ACTGGAGAACGGTGGTTACG
hsa_circ_0002468-covergent	Forward primer	CAGCCAATCAAGAACAACGA
	Reverse primer	GTTGGTGGCAAGCCCTACT
hsa_circ_0004524-covergent	Forward primer	CAGCCAATCAAGAACAACGA
	Reverse primer	TTTATTCTGTTGGTGGCAAGC
hsa_circ_0003823-covergent	Forward primer	TATGTTTCCGGTAGCCCCTA
	Reverse primer	GCCATGCATCATCAATAGCA
hsa_GAPDH-divergent	Forward primer	ACTCCTCCACCTTTGACGC
	Reverse primer	GCTGTAGCCAAATTCGTTGTC
hsa_GAPDH-covergent	Forward primer	GGCCTCCAAGGAGTAAGA
	Reverse primer	GCCCAATACGACCAAATCA
hsa_β_actin	Forward primer	GTGGCCGAGGACTTTGATTG
	Reverse primer	CCTGTAACAACGCATCTCATATT

### Prediction of circRNA-miRNA-mRNA interactions

CircRNA-miRNA interactions were predicted as achieved with use of miRanda software (v3.3a) [42]. Binding capacities between circRNAs and miRNAs were evaluated using total scores and energy scores as calculated using miRanda software, with higher total scores and lower total energies indicating good binding potential. MiRNA target genes were predicted using the StarBase version 2.0 platform which incorporated data from PITA, miRanda, PicTar and TargetScan [43]. The top 5 potential miRNAs and their targeted genes were then selected to map the competing endogenous RNAs (ceRNA) network using Cytoscape software (V.3.2.1) [44]. Additionally, the potential molecular function and involved pathways of these target genes were analyzed in KOBAS 2.0 [38–41].

### Evaluation of dysregulated circRNA coding ability

Most circRNAs originate from protein-coding genes and are mainly located in the cytoplasm, with some even possessing a complete open reading frame (ORF). Although lacking the 5′-cap and 3′-tail, circRNAs containing an internal ribosome entry site (IRES) remain capable of directly recruiting ribosomes to initiate translation [45, 46]. Therefore, IRES and ORF were two primary elements for translation of circRNAs. In the present study, IRES sequence was identified using IRESfinder [47], with an IRES_score ranging from 0 to 1 representing the coding potential. IRES_scores > 0.5 indicate that a circRNA possesses a coding possibility. The ORF was typically determined with use of three sorts of software (CPC [48], CNCI [49] and PFAM [50]) employing standard parameters.

### Cell culture and siRNA transfection

An established HCC cell line, SK-hep1, was purchased from the Cell Bank of Type Culture Collection (Chinese Academy of Sciences, Shanghai, China). This cell line was cultured in DMEM/high glucose medium (Hyclone, Logan, UT, USA) supplemented with 10% fetal bovine serum (FBS) (PAN-Biotek, Aidenbach, Bavaria) and 1% penicillin–streptomycin (Hyclone, Logan, UT, U.S.A.) within a humidified atmosphere of 37 °C containing 5% CO_2._ All siRNAs and a commercial negative siRNA control used in this study were purchased from RiboBio (Guangzhou, China). Transfection of siRNAs was performed using Genmute (SignaGen Laboratories, Rockville, MD, U.S.A.), according to the manufacturers’ instructions.

### CCK-8 assay

Cells were seeded in triplicate into 96-well plates at a density of 2000 cells/well. After incubation for 24 h at 37 °C, 10 μl of CCK-8 solution (Beyotime Biotechnology, Nantong, China) was added to the cell culture medium. This incubation was continued for 1.5 h and the absorbance value at 450 nm was determined using a Spectra Max 250 spectrophotometer (Molecular Devices, Sunnyvale, CA, U.S.A.). Two additional absorbance values at 450 nm were acquired on the third and fifth days after cell seeding.

### Colony formation

HCC cells were suspended and plated into six-well plates at a density of 1000 cells/well. After incubation for 12 days, colonies were fixed with 4% paraformaldehyde (Solarbio, Beijing, China) and stained with Crystal Violet (Beyotime Biotechnology, Nantong, China). The colonies were then photographed and counted.

### Wound-healing assay

HCC cells were incubated in six-well plates (8 × 10^5^ cells/well) until full confluence was achieved. Then, a uniform straight wound was created in the center of the well using a sterile 200 μl micropipette tip. After two washings with PBS, baseline images were obtained using an inverted fluorescent microscope (Carl Zeiss, Jena, Germany). Subsequently, cells were cultured in serum-free medium for 48 h and views at the same location were again captured. Relative migration areas were calculated using Image J software (NIH, Bethesda, MD, U.S.A.).

### Statistical analysis

Statistical analyses were performed using SPSS version 24.0 (IBM SPSS Inc., Chicago, IL, U.S.A.) and results were presented using Prism version 8.0 (GraphPad Software, La Jolla, CA, U.S.A.). Continuous variables were presented as Mean ± standard deviation (SD). Comparisons between groups were analyzed using Student’s t-tests, the Wilcoxon signed rank test, or One-way analysis of variance (one-way ANOVA), as appropriate. Categorical data were analyzed using the Chi-square or Fisher’s exact test. A two-sided P-value of < 0.05 was required for results to be considered as statistically significant. For analyses of differential expressions and enrichments, adjusted *P* values were considered.

## Results

### circRNA expression differences between non-cirrhotic and cirrhotic HCC

**Fig. 1:**
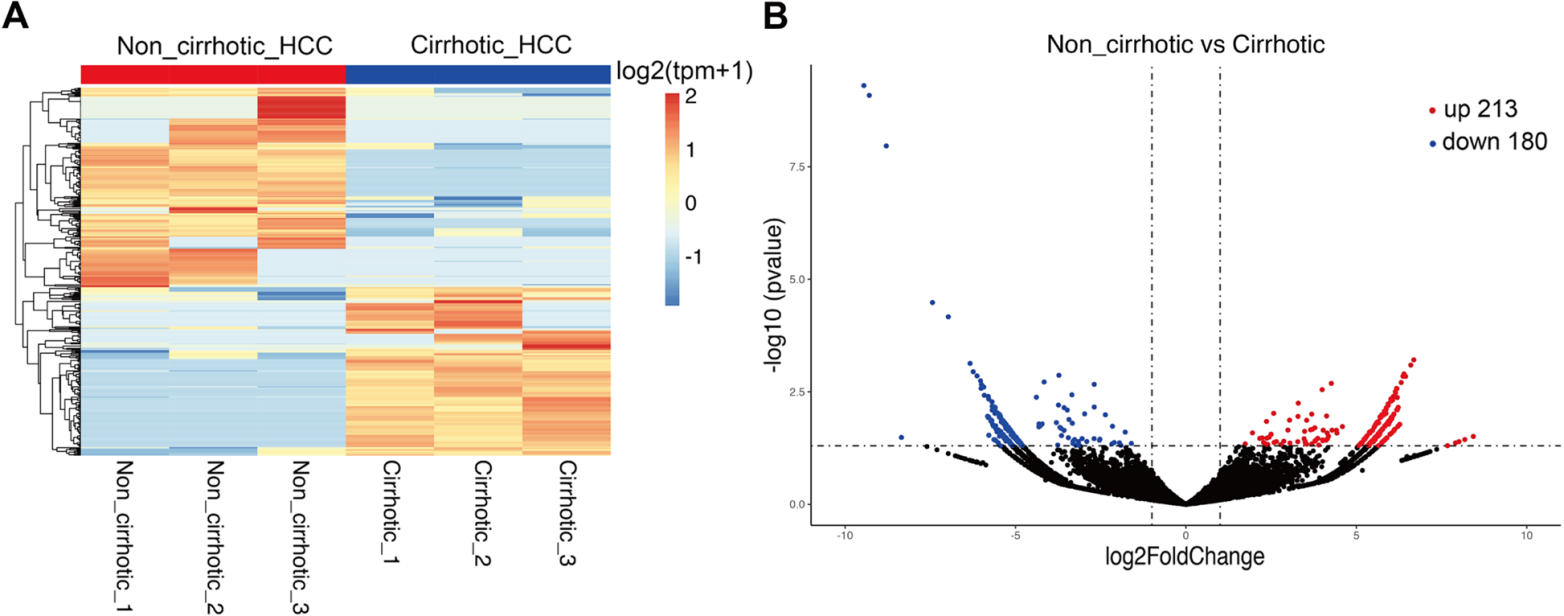
Identification of dysregulated circRNAs between non-cirrhotic and cirrhotic HCC tissues. **A** Hierarchical clustering indicates differences in circRNA expression profiling between the two groups. **B** The volcano plot was showed the expression profiling between the two groups

Based on the histopathological results, six frozen HCC samples (3 from non-cirrhotic and 3 cirrhotic backgrounds) were selected for circRNA-seq. Detailed clinical data of these patients are contained in Table S1. In order to minimize the influence of clinical heterogeneity, some clinical variables including age, gender, HBV-DNA loading and tumor size were matched before sequencing. As shown in Figure S1A and Table S2, a total of 20,334 circRNAs, which were widely distributed in all chromosomes, were detected using circRNA-seq. Among these, 9420 circRNAs were successfully retrieved in circBase, and the remaining 10,914 circRNAs were identified as being novel. Most of these circRNAs ranged from 200 to 600 bp in length and were derived from exons (Figure S1B and S1C). Clustered heatmap results revealed that obvious differences in circRNA expression profiles were present between non-cirrhotic and cirrhotic HCC samples (Fig. 1A and Table S3). According to a differential filtering criteria of (|log2(fold change)|> 1 and *p* < 0.05), 213 significantly up-regulated and 180 significantly down-regulated circRNAs were identified between non-cirrhotic versus cirrhotic HCC tissues (Table S4), as visualized using volcano plots (Fig. 1B). Information of the 10 most prominent dysregulated circRNAs was contained in Table 2.

**Table 2:** Top 10 dysregulated circRNAs

CircRNA_ID	Chrome	Spliced_length	Source_gene	Log2FoldChange	*P*_value	Type
**Up-regulation**						
hsa_circ_0002473	13	200	DNAJC3	6.6833	0.00061767	exon
novel_circ_0013894	1	374	SPATA6	6.5955	0.00080665	exon
novel_circ_0015002	20	330	LBP	6.3962	0.00127001	exon
hsa_circ_0009069	X	265	PHF8	6.3666	0.00143005	exon
novel_circ_0022147	4	340	ADGRL3	6.4435	0.00143922	exon
**Down regulation**						
hsa_circ_0002468	3	396	CEP70	-9.4491	4.98E-10	exon
hsa_circ_0004524	3	470	CEP70	-9.2914	8.20E-10	exon
hsa_circ_0003823	3	522	CEP70	-8.7918	1.09E-08	exon
novel_circ_0003819	12	181	SLCO1B7	-7.4374	3.29E-05	exon
novel_circ_0003806	12	759	SLCO1B7	-6.9721	6.81E-05	exon

### Validation of dysregulated circRNAs by qRT-PCR

**Fig. 2:**
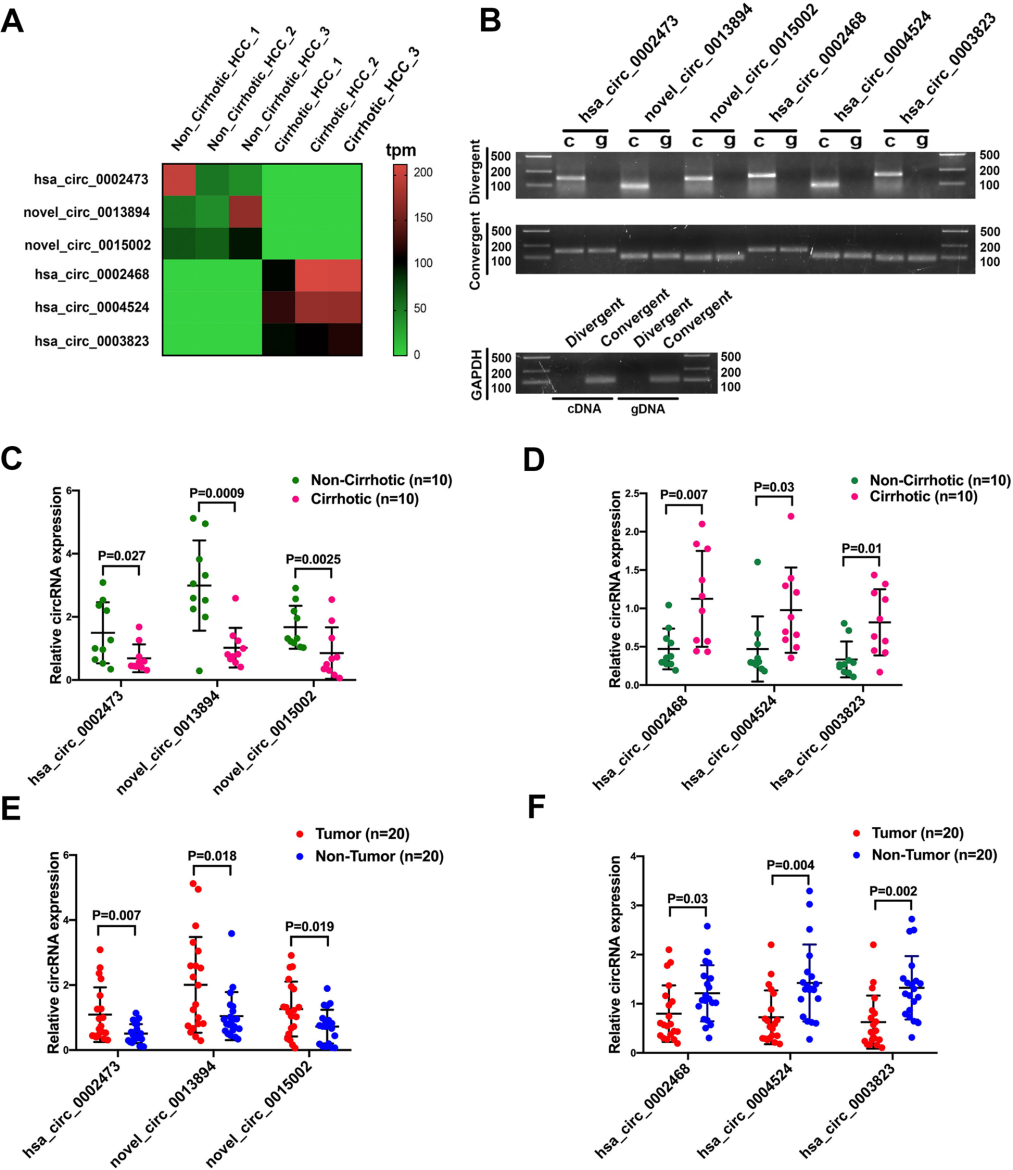
Validation of dysregulated circRNAs. **A** Heat map of the six most prominent dysregulated circRNAs between non-cirrhotic and cirrhotic cases. **B** Agarose gel electrophoresis of PCR products as amplified by divergent and convergent primers of dysregulated circRNAs in cDNA and gDNA with GAPDH used as a reference gene. **C** and **D** Expressions of top three prominent up/down-regulated circRNAs in non-cirrhotic HCC samples as validated using qRT-PCR in 20 HCC samples from patients with or without liver cirrhosis. **E** and **F** Expressions of top three prominent up/down-regulated circRNAs in non-cirrhotic HCC in 20 HCC samples and paired non-cirrhotic tissues. c, cDNA; g, gDNA

To verify these circRNA-seq results, expression levels of the six most prominent dysregulated circRNAs (3 showing maximal up-regulation and 3 maximal down-regulation) (Fig. 2A) were determined in 20 HCC tissues (10 non-cirrhotic and 10 cirrhotic) using qRT-PCR. Detailed clinical data and qRT-PCR results of these patients are presented in Tables S5 and S6. As shown in Fig. 2B and Additional file 9, agarose gel electrophoresis of PCR products revealed that these six circRNAs could only be detected by divergent primers in cDNA, but not gDNA, whereas corresponding linear transcripts could be amplified from both in cDNA and gDNA by convergent primers, with GAPDH serving as the reference gene. These results indicated that all these circRNAs were back-spliced at the post-transcribed stage and existed as a circular structure. The qRT-PCR results were consistent with those of the circRNA-seq data(Fig. 2C and D), demonstrating that the dysregulated circRNAs derived from circRNA-seq were reliable. Additionally, in order to illustrate the potential function of these dysregulated circRNAs, expression levels of these six circRNAs in paired non-tumorous tissues were also detected. The results of this analysis showed that hsa_circ_0002473, novel_circ_0013894 and novel_circ_0015002 were also significantly up-regulated in HCC tissues when compared with that of paired non-tumorous tissues (Fig. 2E), while expression levels of hsa_circ_0002468, hsa_circ_0004524 and hsa_circ_00038 in HCC tissues were significantly lower than that obtained in paired non-tumorous tissues (Fig. 2F). Collectively, these data suggest that an up-regulation of circRNAs in non-cirrhotic HCC may promote the initiation of HCC, while the down-regulation of circRNAs may prevent this initiation.

### Gene Ontology (GO) and Kyoto Encyclopedia of Genes and Genomes (KEGG) analyses for host genes of dysregulated circRNAs

**Fig. 3:**
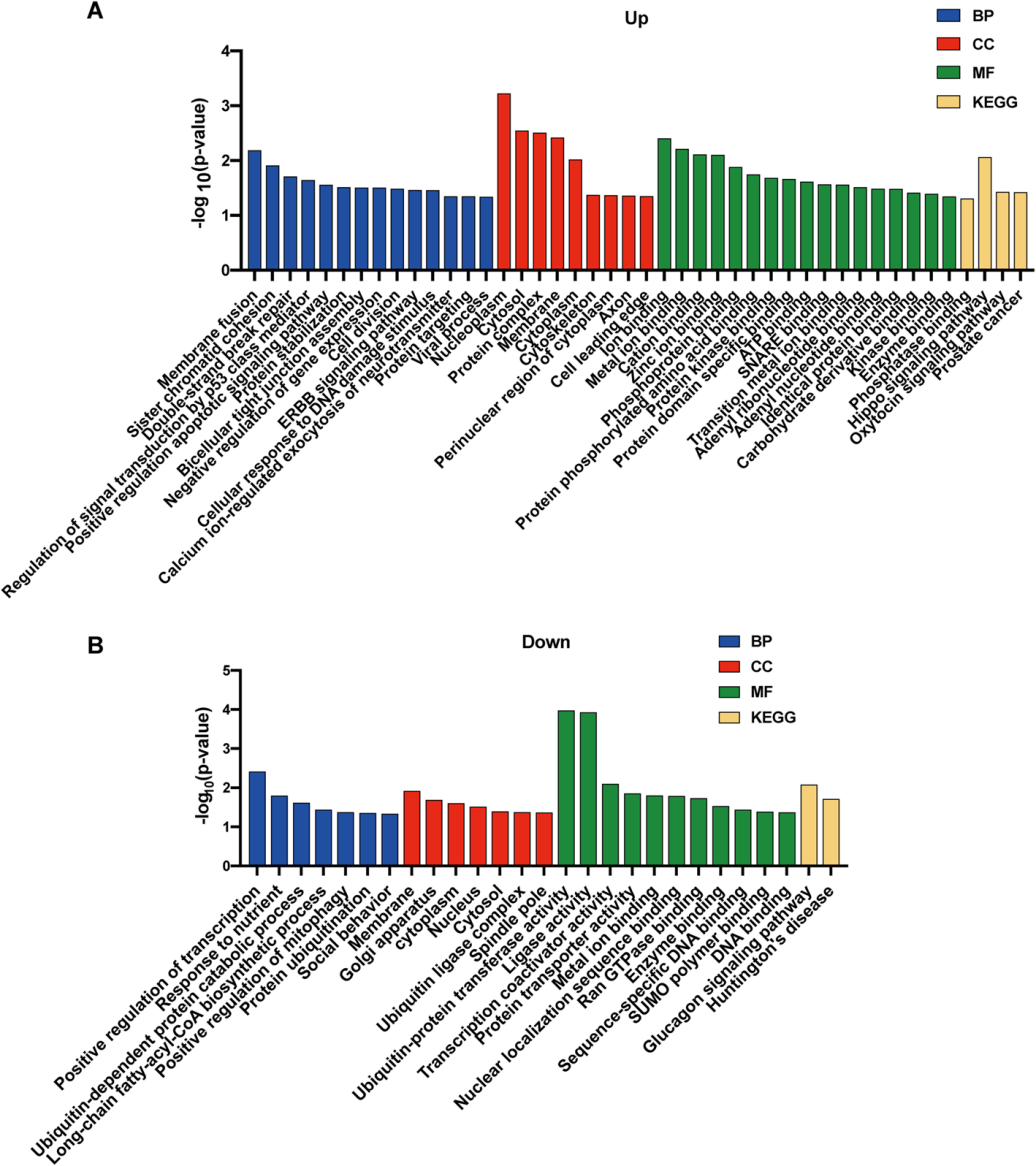
Enrichment analyses of dysregulated circRNAs host genes. **A** GO and KEGG analyses of host genes of up-regulated circRNAs. **B** GO and KEGG analyses of host genes of down-regulated circRNAs

As the production of circRNAs originally involved a back-splicing event of host genes, the biological effects of dysregulated circRNAs may, in part, be predicted by their host genes. Accordingly, in the present study, GO and KEGG enrichment analyses were performed as a mean to predict possible biological functions and signaling pathways of corresponding host genes. Detailed results of these analyses are shown in Table S7 and S8, with items demonstrating a *P*-value < 0.05 visualized in Fig. 3. For the host genes with up-regulated circRNAs (Fig. 3A), it was possible to identify some tumor-related items, including membrane fusion, sister chromatid cohesion, double-strand break repair, Hippo and Oxytocin signaling pathway, and prostate cancer. With regard to host genes with down-regulated circRNAs (Fig. 3B), many tumorigenesis and progression related items were also found to be present, such as positive regulation of transcription, responses to nutrients, ubiquitin-dependent protein catabolic process, and a positive regulation of mitophagy.

### Prediction and visualization of circRNA-miRNA-mRNA relationships

**Fig. 4:**
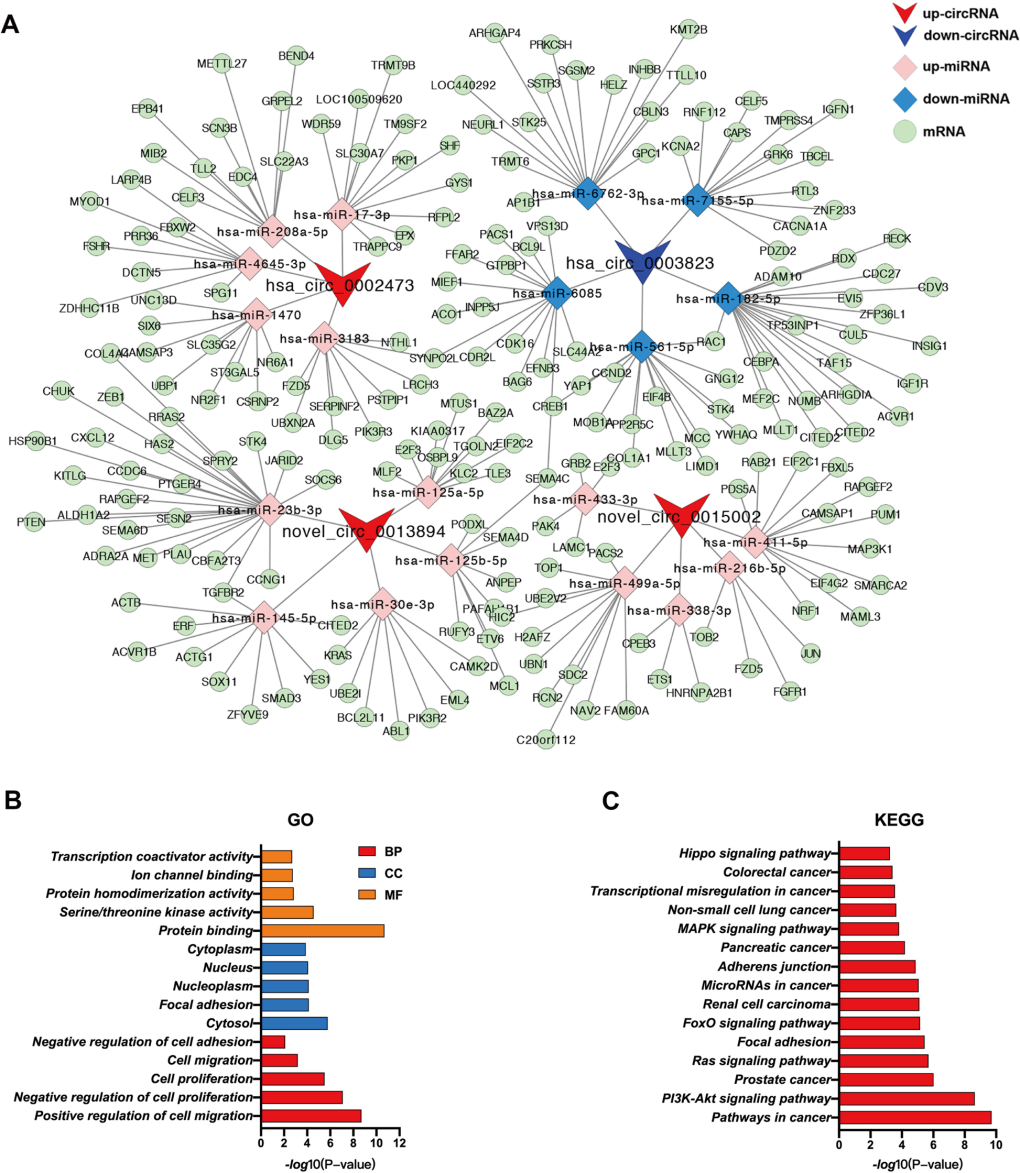
Construction of the circRNA-miRNA-mRNA regulatory network. **A** Interaction network consisting of four circRNAs (three up-regulated and one down-regulated), 20 miRNAs and 214 mRNAs as generated using Cytoscape software (V.3.2.1). **B-C** GO and KEGG analysis were performed to predict the biological function of target mRNAs

The function of ceRNAs represents the earliest and most widely researched mechanisms of circRNAs that were located within the cytoplasm. As a result, circRNAs bind miRNAs and thus prevent their ability to bind and suppress their target mRNAs. In our current study, potential miRNAs of the three most prominent up-regulated and down-regulated circRNAs were predicted using miRanda software. As the top three down-regulated circRNAs, hsa_circ_0002468, hsa_circ_0004524 and hsa_circ_0003823 normally originated from CEP70, and the nucleotide sequences of hsa_circ_0003823 contain hsa_circ_0002468 and hsa_circ_0004524, we utilized this information to predict the candidate miRNAs of hsa_circ_0003823 and top three up-regulated circRNAs (hsa_circ_0002473, novel_circ_0013894 and novel_circ_0015002). The binding capacities between circRNAs and targeted miRNAs were calculated with use of miRanda software and are contained in Table S9. As shown in Fig. 4A, a total of 20 candidate miRNAs and 214 mRNAs were predicted. Among these miRNAs, many were significantly associated with the initiation and progression of various tumors, including miR-182-5p, miR-561-3p, miR-125a-5p, miR-145, miR-23b-3p and miR-30e-3p. Subsequent enrichment analyses also indicated that the downstream mRNAs in the network were significantly related with the biological progression of tumors, including proliferation, migration, differentiation, and focal adhesion (Fig. 4B and C).

### Biological functions of candidate circRNAs as demonstrated in vitro

**Fig. 5:**
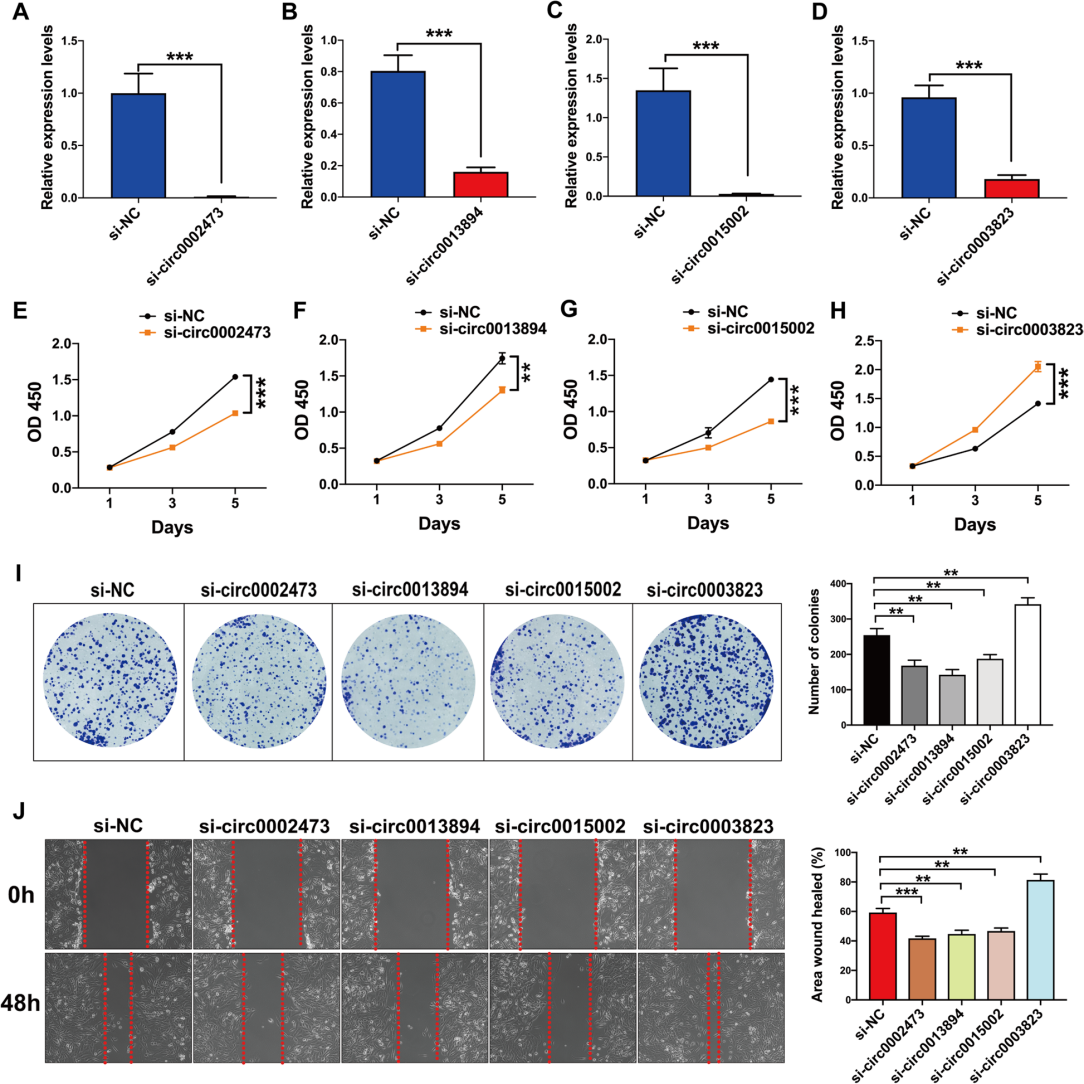
Biological functions of four most prominent dysregulated circRNAs were verified by in vitro experiments. **A-D** Knockdown effect of special siRNAs for these circRNAs was verified by qRT-PCR. **E-H** CCK-8 assay results showed that knockdown of these circRNAs significantly affects the proliferation of HCC cells. **I** Colony formation assay showed the influence of these circRNAs on the survival of HCC cells. **J **Wound healing assay was used to evaluate the biological function of these circRNAs in migration of HCC cells. **P < 0.01, ***P < 0.001. Similar results were obtained in 3 independent experiments

To demonstrate potential functions of these four dysregulated circRNAs, specific siRNAs targeting the back-splice point of circRNAs were synthesized and transfected to SK-Hep1 cells. The qRT-PCR results demonstrated that the expression levels of these circRNAs were clearly knocked down by corresponding siRNAs (Fig. 5A-D). CCK-8 and colony formation assays revealed that the knockdown of circ0002473, circ0013894 or circ0015002 significantly inhibited the proliferation of SK-Hep1 cells, while a decrease in circ0003823 significantly promoted the growth of SK-Hep1 cells (Fig. 5E-I). Results from the wound healing assay suggested that knockdown of circ0002473, circ0013894 or circ0015002 also significantly restrained the migratory ability of HCC cells, while the knockdown of circ0003823 advanced this migration (Fig. 5J). The data used for statistical analyses of these results are contained in Table S10. Taken together, these findings indicate that these circRNAs, as identified using high throughput circRNA-seq, play important roles in HCC progression.

### Coding potential of dysregulated circRNAs

Emerging results have indicated that some circRNAs possess IRES and ORF and can be translated in a cap-independent manner. In the present study, the IRES and ORF of each dysregulated circRNA were predicted. A total of 19 circRNAs possessed both IRES and ORF and were thus considered to have coding possibilities (Table 3). Of these, four circRNAs were significantly upregulated in non-cirrhotic HCC tissues, while the remaining 15 circRNAs were downregulated. These intriguing results on the coding ability and detailed biological functions of these circRNAs will require further investigations to verify their effects.

**Table 3:** Characteristics of dysregulated circRNAs with coding ability

CircRNA_ID	IRES	Chr	Spliced_length	Source_gene	Log2FC	*P*-value
**Up regulation**						
hsa_circ_0001417	0.76	4	1832	ANKRD17	4.181	0.03
hsa_circ_0087840	0.69	9	1003	FSD1L	5.893	0.033
hsa_circ_0000660	0.61	15	1080	MCTP2	6.095	0.023
hsa_circ_0008261	0.59	2	1364	NBAS	5.535	0.033
hsa_circ_0003490	0.59	22	1143	CHEK2	5.713	0.023
**Down regulation**						
hsa_circ_0085086	0.76	8	1738	VPS13B	-5.47	0.03
hsa_circ_0083118	0.75	7	802	LMBR1	-4.94	0.036
hsa_circ_0001675	0.73	7	1132	C1GALT1	-6	0.002
hsa_circ_0018814	0.72	10	1101	PPP3CB	-5.35	0.014
hsa_circ_0001543	0.68	5	1197	NR3C1	-5.5	0.042
hsa_circ_0062984	0.66	22	1022	FBXO7	-5.59	0.007
hsa_circ_0067435	0.63	3	1230	RYK	-4.86	0.044
hsa_circ_0000563	0.62	14	1268	BTBD7	-2.53	0.037
hsa_circ_0039550	0.59	16	1056	RSPRY1	-4.93	0.04
novel_circ_0009512	0.59	17	234	MED13	-6.24	0.001
hsa_circ_0004111	0.59	1	871	GPBP1L1	-4.95	0.036
hsa_circ_0077765	0.56	6	601	RNF217	-5.65	0.007
hsa_circ_0005613	0.54	2	732	RAPH1	-5.45	0.011
hsa_circ_0007108	0.53	X	674	ZFX	-1.9	0.049

## Discussion

In this current study, with use of circRNA-seq, a total of 393 circRNAs demonstrating a significant degree of dysregulation were identified within HCC tissue, with 213 being up-regulated and 180 down-regulated. The reliability of this result was verified as based on results from a larger HCC sample size subjected to qRT-PCR assay.

From results as obtained with in vitro experiments, we also demonstrated that the most prominent dysregulated circRNAs exerted clear effects on the proliferation and migration of HCC cells. Among these differently expressed circRNAs, some have been reported to play important roles in the initiation and progression of other tumors. For example, Hsa_circ_0003823, which is down-regulated in cirrhotic HCC is also significantly down-regulated in breast cancer tissue and may be involved in inhibiting carcinogenesis [51]; and hsa_circ_0043462, derived from ERBB2, is up-regulated in cirrhotic HCC tissues, as well as in various cancers, and thus functions as a tumor promoter. In gallbladder cancer (GBC), circERBB2, located mainly in the nucleus promotes ribosomal DNA transcription and GBC proliferation via the circERBB2-PA2G4-TIFIA regulatory axis [52]. In gastric cancer, expression levels of circERBB2 in preoperative plasma were negatively associated with overall and recurrence-free survival time and, expression levels of circERBB2 in postoperative plasma may be useful in monitoring cancer recurrence [53]. It has also been reported that circERBB2 facilitated the progression of gastric cancer via miR-503/CACUL1 and miR-637/MMP-19 signaling [54]. Although the mechanisms of circRNAs in biological processes were largely different from their host genes, the fact that they share the same chromosome locus and transcription regulation indicates that the biological functions of circRNAs may be partly reflected by their host genes. The enrichment analyses performed in this study revealed that the host genes of dysregulated circRNAs were significantly associated with some well-known cancer related pathways such as membrane fusion, sister chromatid cohesion, double-strand break repair, Hippo and Oxytocin signaling pathway, and prostate cancer. Collectively, these results suggest that the circRNAs identified here likely play important roles in the carcinogenesis of non-cirrhotic HCC patients.

Results from ongoing studies have shown that the majority of circRNAs are mainly distributed within the cytoplasm and may regulate gene expression at post-transcriptional level by sponging miRNAs to promote the expression of miRNA targeted genes. Some circRNAs possess multiple binding sites for a single miRNA. For example, ciRS-7, circZNF91 and circRNA-Sry have 70, 24 and 16 binding sites for miR-7, miR-23b-3p and miR-138, respectively [21, 55]. And, some circRNAs can simultaneously bind different functional miRNAs, like that of cSMARCA5 (hsa_circ_0001445), which suppresses the growth and metastasis of HCC via simultaneously binding miR-17-3p and miR-181b-5p [26]. CircPRKCI functions as a sponge for both miR-545 and miR-589 to promote the carcinogenesis of lung adenocarcinoma [51]. In the present study, the potential for targeted miRNAs consisting of one down-regulated and three up-regulated circRNAs were predicted. Hsa_circ_0003823 is significantly down-regulated in non-cirrhotic HCC, and its candidate miRNAs include miR-182-5p, miR-561-3p, miR-7155-5p, miR-6762-3p and miR-6085. Results from a number of studies have demonstrated that miR-182-5p was significantly up-regulated in HCC tissue and played roles in promoting the initiation, progression and drug resistance of HCC [56, 57]. From a bioinformatic analysis, as based on the TCGA database, miR-561-3p was found to be significantly up-regulated in HCC tissue and served as a predictor for poor overall survival of HCC patients [58]. hsa_circ_0002473, novel_circ_0013894 and novel_circ_0015002 were the three most notable up-regulated circRNAs in non-cirrhotic HCC, and many down-regulated miRNAs in HCC were predicted as their targeted miRNAs, including miR-125a-5p, miR-145, miR-23b-3p and miR-30e-3p. Xu et al. reported that miR-125a-5p was significantly decreased in HCC tissues and cell lines, while an overexpression of miR-125a-5p inhibited the proliferation and induced apoptosis of HCC cell lines by regulating the expression of PTPN1 and MAP3K11 [59]. Noh et al. found that HDAC2 mediated the suppressive effects of miR-145-5p on HCC growth [60]. Gramantieri et al. reported that TP53 decreased the expression of miR-30e-3p in HCC and miR-30e-3p inhibited cell proliferation and invasion of HCC by targeting MDM2, PTEN and CDKN1B/p27 [61]. All these data suggest that sponging miRNAs represent an important avenue for these dysregulated circRNAs in the hepatocarcinogenesis of non-cirrhotic liver.

There are recent reports indicating that some circRNAs can be translated into functional peptides in an IRES-dependent manner. In liver cancer, circβ-catenin produces a novel isoform of β-catenin with the length of 370-amino acids (β-catenin-370aa), and β-catenin-370aa can prevent full-length β-catenin degradation from GSK3β to thus promote activation of the Wnt-pathway [62]. In colon cancer (CC), circPPP1R12A encodes a functional protein (circPPP1R12A-73aa) which promotes the ability for proliferation, migration and invasion of CC via activation of the Hippo-YAP signaling pathway [63]. In glioma, circ-FBXW7 translates a novel functional protein termed as FBXW7-185aa, which inhibits proliferation and cell cycle acceleration by reducing the half-life of c-Myc [64]. In the present study, we also predicted 19 dysregulated circRNAs possessing the potential for coding peptides. However, the function of IRES in recruiting ribosomes will require verification via dual-luciferase assay and the existence of these novel proteins will also need to be verified with use of special antibodies or mass spectrum assays. It will also be necessary to demonstrate their function and underlying mechanisms as achieved through a series of in vivoand in vitro experiments.

## Conclusion

In this study, with use of high-throughput sequencing and qRT-PCR validation, a number of circRNAs were identified to be dysregulated between cirrhotic and non-cirrhotic HCCs. As based on bioinformatic analyses, these dysregulated circRNAs appear to be associated with the tumorigenesis and progression of non-cirrhotic HCC. Moreover, multiple HCC related miRNAs were predicted as downstream targets of these dysregulated circRNAs. And the potential coding possibility of these dysregulated circRNAs was also predicted in this present study.

## Supplementary Information

**Additional file 1:**

**Additional file 2: Table S1. **The clinical data ofpatients who were used for circRNA-seq

**Additional file 3:  Table S2.** The circRNA sequecing data supplied by Novegene

**Additional file 4: Figure S1.**

**Additional file 5: Table S3.** The differently expressed circRNAs achieved by DESeq2 Data used for plot heatmap

**Additional file 6: Table S4.** The differently expressed circRNAs achieved by DESeq2

**Additional file 7: Table S5.** The clinical data ofpatients who were used for validation

**Additional file 8: Table S6.** The qRT-PCR results of circRNA-seq validation

**Additional file 9: Figure 2B.** Original pictures of Figure 2B

**Additional file 10: Table S7.**

**Additional file 11: Table S8.**

**Additional file 12: Table S9.** The binding capacities between circRNAs and miRNAs calculated by miRanda software

**Additional file 13: Table S10.**

## Data Availability

The circRNA-seq data supporting the findings of this study have been deposited in the NCBI Gene Expression Omnibus (accession **GSE193862**).

## Abbreviations

HCC: Hepatocellular carcinoma
qRT-PCR: Quantitative real-time polymerase chain reaction
NAFLD: Non-alcoholic fatty liver disease
EMT: Epithelial-mesenchymal transition
TACE: Transcatheter arterial chemoembolization
ORF: Open reading frame
IRES: Internal ribosome entry site
ROC: Receiver operating characteristic
