# Supplementary material for: High-throughput circular RNA sequencing reveals the profiles of circular RNA in non-cirrhotic hepatocellular carcinoma
Source: BMC Cancer. 2022 Aug 5;22:857. doi: 10.1186/s12885-022-09909-2 (PMC9356431; doi:10.1186/s12885-022-09909-2)
Supplement: Supplementary file 1 — Additional file 1: [file 12885_2022_9909_MOESM1_ESM.pdf]

```
####DESeq2
seqdata<-read.csv("seqdata.csv", row.names = 1)
head(seqdata)
dim(seqdata)
seqdata_1<-seqdata[rowSums(seqdata) != 0,]
dim(seqdata_1)
groupdata<-read.csv("group.csv",stringsAsFactors = T)
groupdata
colnames(seqdata_1) == groupdata$sample

library (DESeq2)

dds <- DESeqDataSetFromMatrix(countData=seqdata_1,
                              colData=groupdata,
                              design=~group)

dds <- DESeq (dds)
res <- results(dds)

head(res)
class(res)
res_1 <- data.frame(res)
class(res_1)
head(res_1)

library(dplyr)
res_1 %>%
  mutate(significant = case_when(
    log2FoldChange >= 1 & pvalue <= 0.05 ~ "up",
    log2FoldChange <= 1 & pvalue <= 0.05 ~ "down",
    TRUE ~ "NOT_CHANGE")) -> res_2

table(res_2$significant)

write.csv(res_2,file="diff_expr_result.csv", quote = F)


###pheatmap

df1<-read.csv("heatmap_data.csv",header=T, row.names = 1,stringsAsFactors = F)
df2<-read.csv("annotation_col.csv",header=T, row.names = 1,stringsAsFactors = F)
df1=t(scale(t(log2(df1+1))))

head(df1)
dim(df1)
head(df2)
library(pheatmap)
anno_colors <- list(group=c(Non_cirrhotic="red",Cirrhotic="blue"))
pheatmap(df1,cluster_cols=F,show_colnames = T, fontsize=20, show_rownames =
F)
```

```
pheatmap(df1,cluster_cols=F,show_colnames = T, fontsize=15, show_rownames =
F,annotation_col = df2,
         annotation_colors = anno_colors)

library(export)
graph2pdf(file="heatmap.pdf")



####volcano

allDiff<-read.csv("diff_expr_result.csv", row.names = 1)
data <- allDiff

library(ggplot2)
data$significant <- as.factor(data$pvalue<0.05 & abs(data$log2FoldChange) > 1)
ggplot(data=data, aes(x=log2FoldChange, y =-log10(pvalue),color
=significant,size=0.1)) +
  geom_point(size=1) +
  scale_color_manual(values =c("black","red"))+
  geom_hline(yintercept = -log10(0.05),lty=4,lwd=0.6,alpha=0.8)+
  geom_vline(xintercept = c(1,-1),lty=4,lwd=0.6,alpha=0.8)+
  theme_bw()+
  theme(panel.border = element_blank(),
        panel.grid.major = element_blank(),
        panel.grid.minor = element_blank(),
        axis.line = element_line(colour = "black"))+
  labs(title="Non_cirrhotic vs Cirrhotic", x="log2FoldChange",y="-log10 (pvalue)")+
  theme(plot.title = element_text(hjust = 0.5,size=20),
        axis.title.x = element_text(size = 15),
        axis.title.y = element_text(size = 15))+
  xlim(-10,10)

library(export)
graph2pdf(file="volcano.pdf")
```
