# Supplementary material for: High-throughput circular RNA sequencing reveals the profiles of circular RNA in non-cirrhotic hepatocellular carcinoma
Source: BMC Cancer. 2022 Aug 5;22:857. doi: 10.1186/s12885-022-09909-2 (PMC9356431; doi:10.1186/s12885-022-09909-2)
Supplement: Supplementary file 2 — Additional file 2: Table S1. The clinical data ofpatients who were used for circRNA-seq [file 12885_2022_9909_MOESM2_ESM.docx]

**Table S1. The clinical data of patients who were used for circRNA-seq**

| Patient | Ishak  score | Gender | Age (year) | AFP (ng/mL) | Child-Pugh | Tumor size (cm) | BCLC stage | Capsule Invasion | MVI | Differentiation |
| --- | --- | --- | --- | --- | --- | --- | --- | --- | --- | --- |
| Non_cirrhosis_1 | 2 | Male | 69 | <400 | A | 4.5 | B | No | No | High |
| Non_cirrhosis_2 | 2 | Male | 49 | ≥400 | A | 7.8 | C | No | No | Low |
| Non_cirrhosis_3 | 2 | Male | 50 | <400 | A | 5 | B | No | Yes | Low |
| Cirrhosis_1 | 6 | Male | 61 | <400 | A | 4.3 | B | Yes | No | Low |
| Cirrhosis_2 | 6 | Male | 47 | ≥400 | A | 7.7 | C | No | Yes | High |
| Cirrhosis_3 | 6 | Male | 50 | <400 | A | 5.1 | B | No | No | High |

AFP, alpha-fetoprotein; BCLC, Barcelona Clinic Liver Cancer staging system; MVI, microvascular invasion.
