# Supplementary figures and images for: High-throughput circular RNA sequencing reveals the profiles of circular RNA in non-cirrhotic hepatocellular carcinoma
Source: BMC Cancer. 2022 Aug 5;22:857. doi: 10.1186/s12885-022-09909-2 (PMC9356431; doi:10.1186/s12885-022-09909-2)

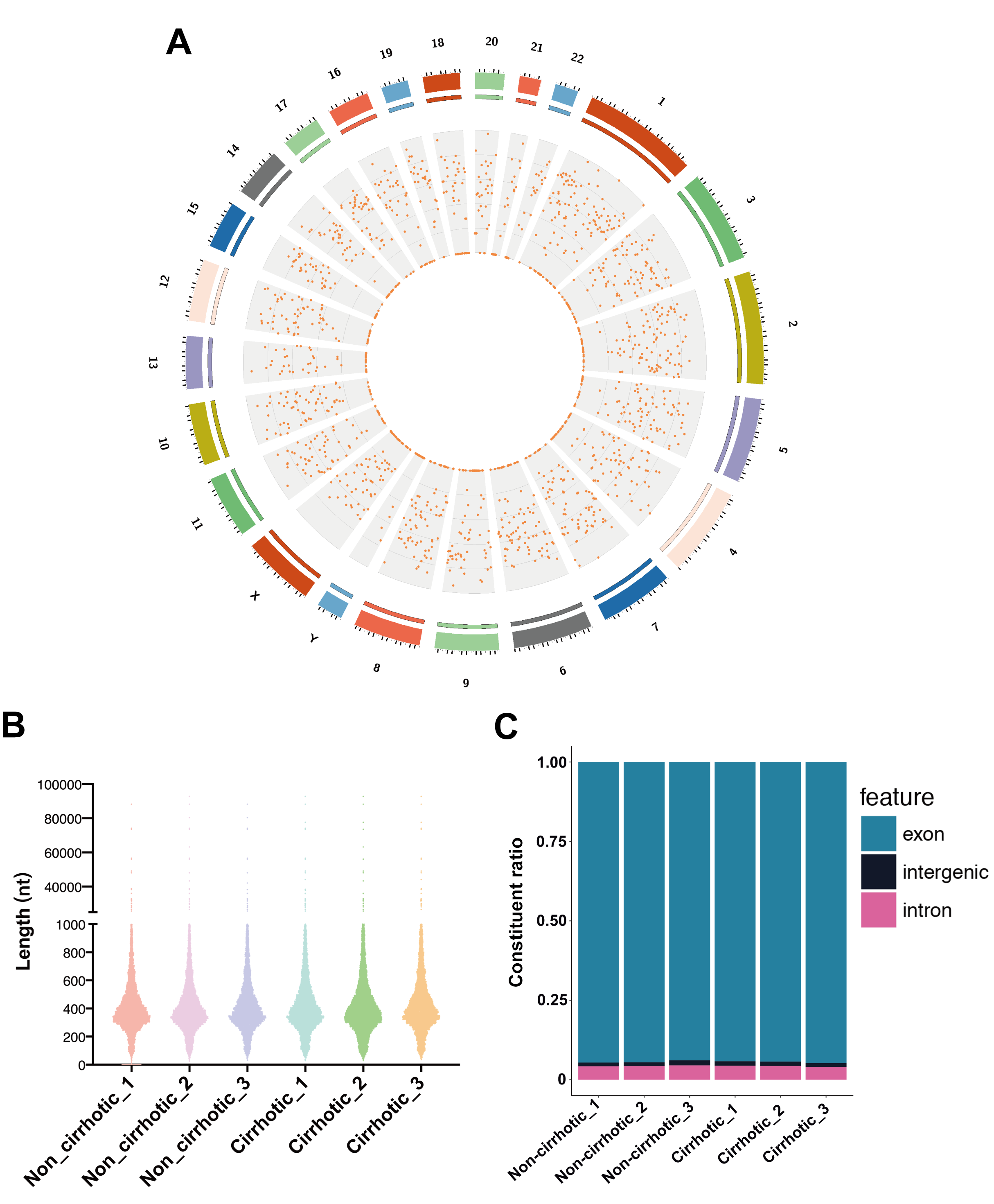

Supplement: Supplementary file 4 — Additional file 4: Figure S1. [file 12885_2022_9909_MOESM4_ESM.tif]

**Original pictures of Figure 2B**

(1) Gel of divergent primers


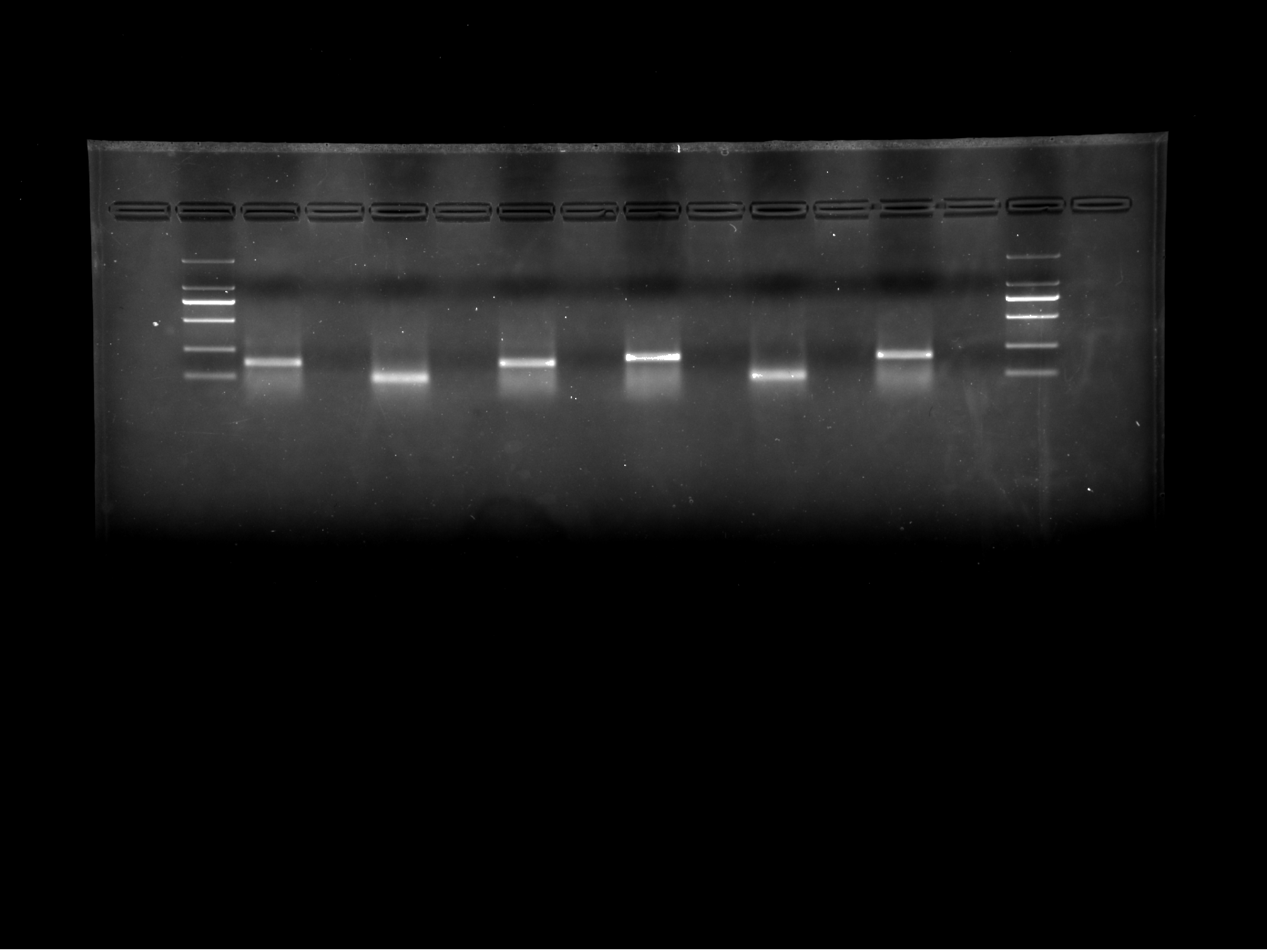


(2) Gel of convergent primers and GAPDH


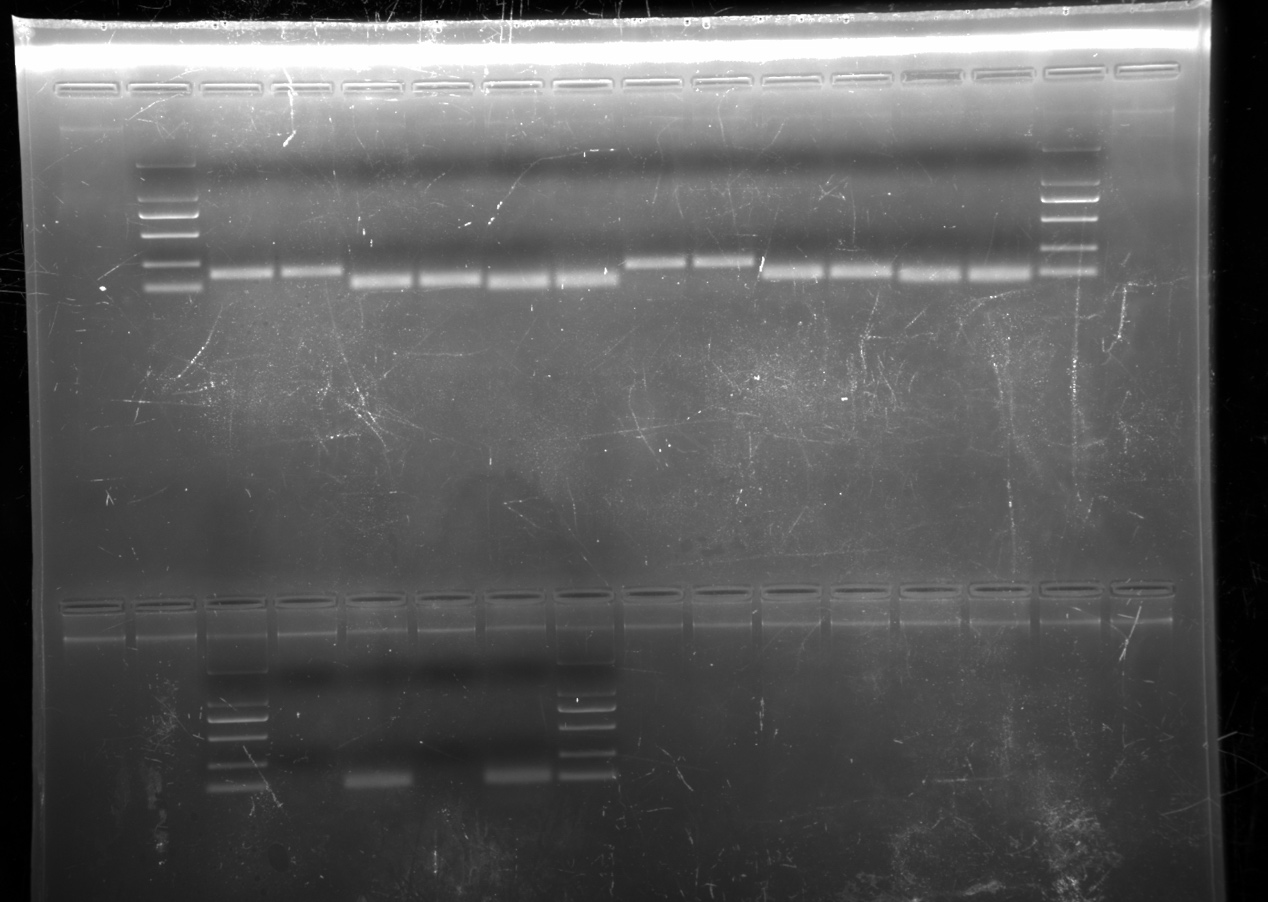

Supplement: Supplementary file 8 — Additional file 8: Table S6. The qRT-PCR results of circRNA-seq validation [file 12885_2022_9909_MOESM8_ESM.docx]
