# Supplementary material for: High-throughput circular RNA sequencing reveals the profiles of circular RNA in non-cirrhotic hepatocellular carcinoma
Source: BMC Cancer. 2022 Aug 5;22:857. doi: 10.1186/s12885-022-09909-2 (PMC9356431; doi:10.1186/s12885-022-09909-2)
Supplement: Supplementary file 7 — Additional file 7: Table S5. The clinical data ofpatients who were used for validation [file 12885_2022_9909_MOESM7_ESM.docx]

**Table S5. The clinical data of patients who were used for validation**

| Group | Ishak score | Gender | Age (year) | AFP (ng/mL) | Child-Pugh | Tumor size (cm) | BCLC stage | Capsule Invasion | MVI | Differentiation |
| --- | --- | --- | --- | --- | --- | --- | --- | --- | --- | --- |
| Non_cirrhosis | 2 | Male | 69 | <400 | A | 4.5 | B | No | No | High |
| Non_cirrhosis | 2 | Male | 49 | ≥400 | A | 7.8 | C | No | No | Low |
| Non_cirrhosis | 2 | Male | 50 | <400 | A | 5 | B | No | Yes | Low |
| Non_cirrhosis | 3 | Male | 45 | <400 | A | 11.6 | B | Yes | No | Low |
| Non_cirrhosis | 3 | Female | 44 | <400 | A | 11.9 | B | Yes | No | Low |
| Non_cirrhosis | 3 | Male | 70 | <400 | A | 10 | B | Yes | No | Low |
| Non_cirrhosis | 3 | Male | 52 | ≥400 | A | 4.8 | B | No | Yes | Low |
| Non_cirrhosis | 3 | Male | 64 | <400 | A | 7.7 | B | No | Yes | High |
| Non_cirrhosis | 3 | Female | 73 | <400 | A | 8 | B | No | No | High |
| Non_cirrhosis | 3 | Male | 42 | ≥400 | A | 3 | B | Yes | No | High |
| cirrhosis | 6 | Male | 52 | ≥400 | A | 4 | B | No | Yes | High |
| cirrhosis | 6 | Male | 45 | ≥400 | A | 6 | B | No | Yes | Low |
| cirrhosis | 6 | Male | 49 | ≥400 | A | 4.2 | B | Yes | Yes | High |
| cirrhosis | 6 | Male | 49 | <400 | A | 3.4 | B | No | No | Low |
| cirrhosis | 6 | Male | 61 | <400 | A | 4.3 | B | Yes | No | High |
| cirrhosis | 6 | Male | 65 | <400 | A | 6 | B | Yes | No | Low |
| cirrhosis | 6 | Male | 46 | ≥400 | A | 9 | C | No | No | Low |
| cirrhosis | 6 | Male | 33 | <400 | A | 12.3 | C | No | Yes | High |
| cirrhosis | 6 | Male | 45 | ≥400 | A | 10.5 | C | Yes | No | High |
| cirrhosis | 6 | Male | 36 | <400 | A | 11.2 | C | No | Yes | High |

AFP, alpha-fetoprotein; BCLC, Barcelona Clinic Liver Cancer staging system; MVI, microvascular invasion.
